# Epidemiological Profile of Uropathogenic Extended‐Spectrum Beta‐Lactamase‐Producing Enterobacteriaceae

**DOI:** 10.1155/ipid/4689685

**Published:** 2026-05-27

**Authors:** Soufyane Yassara, Samira Jaouhar, Khadija Benyahia, Mohammed Sbiti, Khadija Bekhti

**Affiliations:** ^1^ Laboratory of Microbial Biotechnology & Bioactive Molecules, Faculty of Sciences and Techniques, Sidi Mohamed Ben Abdellah University, Fez, PB 2202, Morocco, usmba.ac.ma; ^2^ Higher Institute of Nursing and Health Professions of Fez, Annex Meknes, Regional Directorate of Health Fes-Meknes, Street Omar El Farouk Hamria PB 267, Meknes, 50000, Morocco; ^3^ Health Sciences and Technologies Laboratory, Higher Institute of Health Sciences, Hassan First University, Casablanca Road km 3.5 B.P 555, Settat, Morocco, uh1.ac.ma; ^4^ Bacteriology Department, Moulay Ismaïl Military Hospital, Faculty of Medicine and Pharmacy and Dentistry, Sidi Mohamed Ben Abdellah University, Fez, PB 2202, Morocco, usmba.ac.ma

**Keywords:** antimicrobial resistance, Enterobacteriaceae, ESBL, retrospective study, urinary tract infections

## Abstract

**Background:**

The global spread of extended‐spectrum beta‐lactamase‐producing Enterobacteriaceae (ESBL‐E) represents a major challenge for the management of urinary tract infections (UTIs), limiting therapeutic options and increasing the risk of treatment failure. This study aimed to characterize the epidemiological profile and antimicrobial resistance patterns of ESBL‐producing uropathogenic *Enterobacteriaceae* in a Moroccan hospital setting.

**Methods:**

A retrospective descriptive study was conducted in the bacteriology department of the Moulay Ismail Military Hospital in Meknes, Morocco, over a 2‐year period (2021–2023). Data were extracted from archived urine cytobacteriological examinations. Bacterial identification was performed using chromogenic media and biochemical methods, while antimicrobial susceptibility testing was carried out using the disk diffusion method according to CA‐SFM guidelines. ESBL production was detected using the double‐disk synergy test.

**Results:**

Among 9867 urine samples analyzed, 1676 cases of UTIs were identified, corresponding to a prevalence of 17%. Enterobacteriaceae accounted for 56.8% of isolates, among which ESBL‐producing strains represented the majority of multidrug‐resistant bacteria. *Escherichia coli* was the predominant species (69.9%), followed by *Klebsiella pneumoniae* (17.6%) and *Klebsiella oxytoca* (11%). High resistance rates were observed to third‐generation cephalosporins (up to 96% for ceftriaxone) and fluoroquinolones (93% for ciprofloxacin). In contrast, carbapenems, amikacin, and fosfomycin retained good activity, with resistance rates below 10%.

**Conclusions:**

This study highlights the increasing burden of ESBL‐producing Enterobacteriaceae in UTIs and the alarming levels of resistance to commonly used antibiotics. These findings underscore the need for continuous surveillance, rational antibiotic use, and strengthened antimicrobial stewardship programs to limit the spread of multidrug‐resistant bacteria and preserve the effectiveness of available treatments.

## 1. Introduction

Urinary tract infections (UTIs) are among the most common bacterial infections worldwide and account for a substantial burden of morbidity, healthcare utilization, and antibiotic consumption [1, 2]. They are mainly caused by uropathogens belonging to the Enterobacteriaceae family, particularly *Escherichia coli* and *Klebsiella pneumoniae,* although other Gram‐negative and Gram‐positive organisms may also be involved [2, 3].

The emergence and dissemination of extended‐spectrum beta‐lactamase (ESBL)‐producing Enterobacteriaceae have markedly complicated the management of UTIs because these organisms are resistant to most penicillins, third‐generation cephalosporins, and monobactams, and frequently display co‐resistance to other antibiotic classes [4, 5]. This multidrug‐resistant (MDR) phenotype is often mediated by plasmids and other mobile genetic elements that facilitate the rapid spread of resistance determinants within healthcare settings and the community [6].

The World Health Organization has identified resistant Enterobacteriaceae among the bacterial pathogens of highest public health importance, and antimicrobial resistance is now recognized as a major global health threat associated with excess mortality, prolonged hospital stays, and increased healthcare costs [7, 8]. In addition to microbiological factors, several host‐related conditions such as advanced age, previous antibiotic exposure, diabetes mellitus, recurrent UTIs, and healthcare contact may increase the risk of infection with ESBL‐producing organisms [9].

At the Moulay Ismail Military Hospital (MIMH) in Meknes, earlier retrospective studies documented the epidemiological evolution of uropathogenic Enterobacteriaceae and highlighted a progressive increase in resistance over time [10, 11]. Building on this local background, the present study aimed to characterize the epidemiological profile and antimicrobial resistance patterns of ESBL‐producing uropathogenic Enterobacteriaceae isolated from urine samples processed in the bacteriology laboratory during the 2021‐2023 study period.

## 2. Methods

### 2.1. Study Design and Setting

This retrospective, descriptive study was conducted in the bacteriology department of the MIMH in Meknes using the archive of the laboratory. It included urine cytobacteriological examinations (UCBE) processed over a 2‐year period (2021–2023).

### 2.2. Ethical Considerations

This study used only archived and anonymized laboratory records. No direct patient contact, intervention, or modification of routine care was involved. In accordance with the institutional framework applicable to retrospective analyses of anonymized data, formal ethical approval and informed consent were not required. All data were handled confidentially and in accordance with the principles of the Declaration of Helsinki.

### 2.3. Inclusion and Exclusion Criteria

All urine samples with significant bacteriuria were eligible for inclusion, regardless of patient age, sex, hospitalization status, or consultation setting (inpatient, outpatient, or emergency department). Duplicate isolates from the same patient were excluded in order to avoid overrepresentation of recurrent samples.

### 2.4. Sample Collection

Urine specimens were collected in sterile containers before initiation of antibiotic therapy and were processed within 2 hours of receipt to preserve sample quality and ensure reliable microbiological analysis.

### 2.5. Laboratory Analysis

#### 2.5.1. Microscopy and Culture

Routine urinary cytobacteriological examination included microscopic analysis using Fast Read to detect leukocytes, erythrocytes, epithelial cells, casts, and crystals. Uroculture was performed on bromocresol purple (BCP) agar as the primary isolation medium, while blood agar was used when the presence of chain‐forming cocci was suspected.

#### 2.5.2. Interpretation Criteria

Results were interpreted according to Kass criteria. UTI was defined by leukocyturia > 10^4^/mL associated with bacteriuria > 10^5^ CFU/mL. For some recognized uropathogens, including *Escherichia coli* and *Staphylococcus saprophyticus*, a lower threshold of 10^3^ CFU/mL was considered clinically significant.

#### 2.5.3. Bacterial Identification

Bacterial identification was performed using UriSelect chromogenic medium (Bio‐Rad) and completed, when necessary, by biochemical identification with BioMérieux API systems (API 20E, API 20NE, API Staph, and API Strep). Latex agglutination was used for the identification of selected species‐specific antigens.

#### 2.5.4. Antibiotic Susceptibility Testing

Antibiotic susceptibility testing was performed using the Mueller–Hinton agar disk diffusion method. Results were interpreted following guidelines from the Antibiogram Committee of the French Microbiology Society (AC‐FMS) [12].

#### 2.5.5. ESBL Detection

ESBL production was detected by the double‐disk synergy test using amoxicillin–clavulanic acid in combination with cefotaxime and/or ceftriaxone. A characteristic enhancement of the inhibition zone (“champagne cork” image) was considered indicative of ESBL production. Additional testing with ceftazidime, aztreonam, and cefepime was performed to further describe the associated resistance profile. For isolates showing reduced susceptibility to ertapenem, carbapenemase production was investigated using the modified Hodge test, which was the routine phenotypic method available in the laboratory during the study period. No alternative confirmatory assays, such as Carba NP, mCIM/eCIM, or molecular detection methods, were systematically performed.

### 2.6. Data Management and Analysis

Data were extracted from the computerized bacteriology laboratory database and entered into Microsoft Excel 2019 for analysis. Descriptive statistics were used to summarize the data, including frequencies, percentages, and averages, and the results were presented in tables and figures.

## 3. Results

### 3.1. Burden of UTI and MDR

**FIGURE 1:**
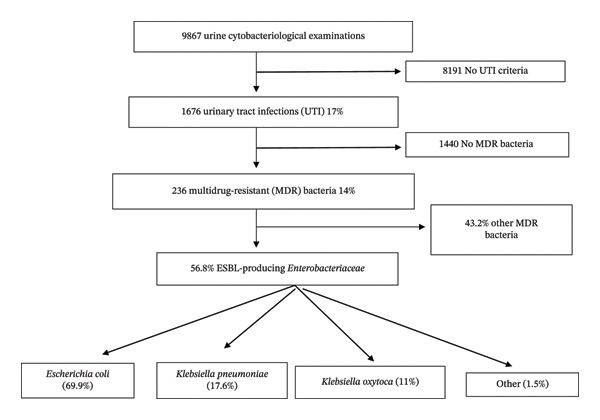
Flowchart showing the different results found in this study.

During the study period, the bacteriology laboratory processed 9867 urine cytobacteriological examinations. Among them, 1676 fulfilled the microbiological criteria for UTI, corresponding to a prevalence of 17.0% (Figure 1). Of these positive cultures, 236 isolates (14.1%) were classified as MDR.

### 3.2. Distribution of MDR Isolates

Among MDR isolates, ESBL‐producing *Enterobacteriaceae* were the most frequent group, accounting for 56.8% of cases, followed by methicillin‐resistant *Staphylococcus aureus* (17.4%) and carbapenemase‐producing Enterobacteriaceae (15.2%). Lower proportions were observed for imipenem‐resistant *Acinetobacter baumannii*, imipenem‐resistant *Pseudomonas aeruginosa*, and ceftazidime‐resistant *Pseudomonas aeruginosa* (Table 1).

**TABLE 1:** Multidrug‐resistant (MDR) rates by bacterial species.

MDR	Numbers	Percentage (%)
ESBL	134	56.8
MRSA	41	17.4
CPE	36	15.2
IRAB	12	5.1
IRPA	7	3
CRPA	6	2.5

Abbreviations: CPE, carbapenemase‐producing Enterobacteriaceae; CRPA, ceftazidime‐resistant *Pseudomonas aeruginosa*; IRAB, imipenem‐resistant *Acinetobacter baumannii*; IRPA, imipenem‐resistant *Pseudomonas aeruginosa*; MRSA, methicillin‐resistant *Staphylococcus aureus*.

### 3.3. Distribution of ESBL Isolates by Sex and Hospital Ward

Among ESBL‐producing Enterobacteriaceae, 68% of isolates were recovered from male patients and 32% from female patients. Regarding hospital distribution, the highest proportion was observed in the urology department (72%), followed by the intensive care unit (12%), visceral surgery (9%), and the emergency department (7%).

### 3.4. Species Distribution and Antimicrobial Resistance Profile

Escherichia coli was the predominant ESBL‐producing species, representing 69.9% of isolates. It was followed by *Klebsiella pneumoniae* (17.6%) and *Klebsiella oxytoca* (11.0%).

**FIGURE 2:**
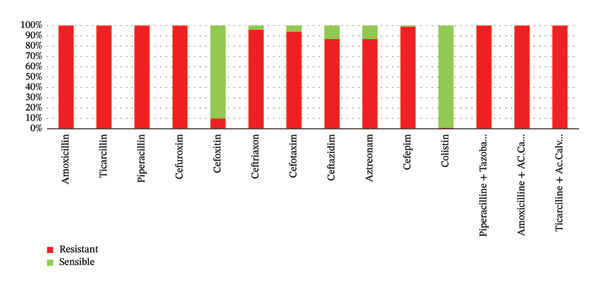
Study of ESBL‐E resistance/sensitivity to the beta‐lactam antibiotics tested.

**FIGURE 3:**
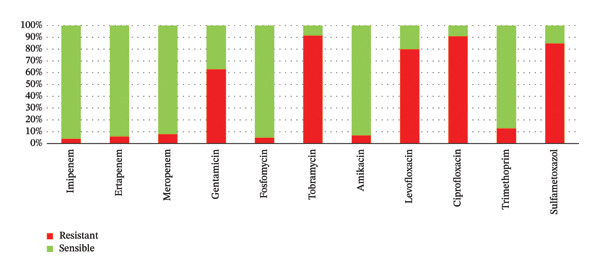
Associated resistance profile of ESBL‐E to the other antibiotics tested.

The antimicrobial susceptibility profile of ESBL‐producing Enterobacteriaceae showed very high resistance to extended‐spectrum beta‐lactams, including ceftriaxone (96%), ceftazidime (87%), aztreonam (87%), and cefepime (81%) (Figure 2). Fluoroquinolone resistance was also high, reaching 91% for ciprofloxacin and 80% for levofloxacin. Moderate resistance was observed for gentamicin (63%) and tobramycin (66%). By contrast, low resistance rates were found for amikacin (7%), imipenem (4%), ertapenem (6%), fosfomycin (5%), and colistin (1%), indicating that these agents remain among the most active options against the isolates analyzed in this study (Figure 3).

## 4. Discussion

UTIs remain one of the most frequent bacterial infections encountered in both hospital and community settings. Their microbiological profile is dominated by Enterobacteriaceae, particularly *E. coli* and *K. pneumoniae*, but the increasing circulation of MDR strains has made empirical treatment progressively more difficult [13]. In this context, local epidemiological surveillance is essential because the prevalence of resistant uropathogens varies substantially according to region, healthcare setting, and antibiotic‐use practices.

In the present study, the overall prevalence of microbiologically confirmed UTI was 17.0%. This value differs from the incidence figures reported in other contexts, such as catheter‐associated UTIs in India or postoperative UTIs in the United States [14, 15], illustrating the heterogeneity of study populations and diagnostic approaches. Such variation also reflects differences in healthcare structures, patient characteristics, and sample selection strategies.

MDR isolates represented 14.1% of all positive cultures, a proportion close to the rates previously reported in Rabat (13%) and Oujda (16.8%) [16, 17]. Although methodological differences should be considered, these data support the idea that antimicrobial resistance among uropathogens remains a sustained and clinically relevant problem in Morocco. In our series, ESBL‐producing Enterobacteriaceae constituted the leading MDR phenotype, accounting for 56.8% of all MDR isolates.

The predominance of ESBL‐producing isolates in our study appears markedly higher than that reported in earlier work conducted at the same institution by Lahlou and by Sbiti [10, 11], suggesting a progressive increase over time. Comparable studies from Marrakech and Rabat also reported lower proportions than those observed here [15, 17], whereas a higher rate was described in Oujda [16]. This interstudy variability may be explained by differences in patient recruitment, referral patterns, prior antibiotic exposure, underlying comorbidities, and local infection‐control practices.

The sex distribution in our study showed a predominance of male patients among ESBL cases. This finding contrasts with many epidemiological reports in which women are more frequently affected by UTIs [17, 18]. In our setting, however, the hospital population includes a large number of active or retired military men, and urine cytobacteriological testing is frequently requested in the context of urological disorders, particularly among older men with prostatic disease. This local case mix may explain the observed male predominance.

Regarding ward distribution, the concentration of ESBL isolates in the urology department is consistent with the clinical nature of the institution and with previous local data [12]. The predominance of *E. coli*, followed by *K. pneumoniae* and *K. oxytoca*, also agrees with the classical microbiology of UTIs and with earlier Moroccan studies [11, 16, 19, 20]. Collectively, these observations confirm that ESBL‐producing *E. coli* remains the leading pathogen in resistant urinary infections.

The resistance profile observed in this study is of particular clinical concern. ESBL isolates displayed very high resistance to third‐ and fourth‐generation cephalosporins and to aztreonam, which is expected given the enzymatic activity of ESBL*s*. High co‐resistance to fluoroquinolones was also documented, further narrowing oral treatment options. This pattern is consistent with the well‐described co‐selection of resistance determinants carried on transmissible plasmids, including genes associated with quinolone resistance and resistance to multiple antibiotic families [21–23].

Conversely, resistance remained low for amikacin, carbapenems, fosfomycin, and colistin. These results are in line with previous reports suggesting that such agents may still retain useful activity against ESBL‐producing urinary isolates, particularly in severe infections or when oral options are limited [24, 25]. Nevertheless, their preservation requires careful stewardship, because increasing use inevitably generates selection pressure and may accelerate the emergence of additional resistance mechanisms.

A methodological point that deserves caution is the detection of carbapenemase production by the modified Hodge test. Although this assay was part of routine laboratory practice during the study period, it is now considered less reliable than newer phenotypic methods, particularly for the detection of metallo‐beta‐lactamases. Consequently, the proportion of carbapenemase‐producing Enterobacteriaceae reported in this study should be interpreted in light of the methodological context in which the data were generated.

From a clinical and public health perspective, the high burden of ESBL‐producing Enterobacteriaceae highlighted by this study underlines the need for continuous microbiological surveillance, prudent antibiotic prescribing, and reinforcement of infection‐prevention measures. Local treatment protocols should be regularly updated according to institutional resistance data, and empirical therapy should be reassessed once susceptibility results become available.

## 5. Limitations

This study has several limitations. First, its retrospective design depends on the completeness and quality of archived laboratory records. Second, carbapenemase detection relied on the modified Hodge test, without systematic confirmation by newer phenotypic or molecular methods. Third, no molecular characterization of ESBL or carbapenemase genes was performed. Despite these limitations, the study provides useful epidemiological data on the burden and resistance profile of uropathogenic ESBL‐producing Enterobacteriaceae in our setting.

## 6. Conclusion

This retrospective study highlights the significant burden of extended‐spectrum beta‐lactamase‐producing Enterobacteriaceae (ESBL‐E) in UTIs within a Moroccan hospital setting. The high resistance rates observed, particularly to third‐generation cephalosporins and fluoroquinolones, considerably limit empirical treatment options and raise concerns about therapeutic failures.

The preserved susceptibility to carbapenems, amikacin, and fosfomycin suggests that these antibiotics remain effective alternatives; however, their increased use may contribute to the emergence of further resistance. These findings emphasize the urgent need for optimized antimicrobial stewardship strategies, improved diagnostic approaches, and continuous epidemiological surveillance.

Future studies incorporating molecular characterization of resistance mechanisms and more advanced detection methods are essential to better understand resistance dynamics and guide effective clinical management. Strengthening infection‐control measures and promoting rational antibiotic use remain key priorities to mitigate the spread of MDR bacteria.

## Funding

This work was carried out with the support of the National Center for Scientific and Technical Research (CNRST), under the “PhD‐Associate Scholarship‐PASS” program.

## Conflicts of Interest

The authors declare no conflicts of interest.

## Data Availability

The data that support the findings of this study are available from the corresponding author upon request.
